# Expression of group II and III mGluRs in the carotid body and its role in the carotid chemoreceptor response to acute hypoxia

**DOI:** 10.3389/fphys.2022.1008073

**Published:** 2022-09-22

**Authors:** Chenlu Zhao, Chaohong Li, Baosheng Zhao, Yuzhen Liu

**Affiliations:** ^1^ Henan Key Laboratory of Neurorestoratology, Life Science Research Center, The First Affiliated Hospital of Xinxiang Medical University, Weihui, Henan, China; ^2^ Department of Thoracic Surgery, The First Affiliated Hospital of Xinxiang Medical University, Weihui, Henan, China

**Keywords:** carotid body, group II mGluRs, group III mGluRs, carotid sinus nerve, hypoxia

## Abstract

The carotid body (CB) contributes significantly to oxygen sensing. It is unclear, however, whether glutamatergic signaling is involved in the CB response to hypoxia. Previously, we reported that ionotropic glutamate receptors (iGluRs) and multiple glutamate transporters are present in the rat CB. Except for iGluRs, glutamate receptors also include metabotropic glutamate receptors (mGluRs), which are divided into the following groups: Group I (mGluR1/5); group II (mGluR2/3); group III (mGluR4/6/7/8). We have studied the expression of group I mGluRs in the rat CB and its physiological function response to acute hypoxia. To further elucidate the states of mGluRs in the CB, this study’s aim was to investigate the expression of group II and III mGluRs and the response of rat CB to acute hypoxia. We used reverse transcription-polymerase chain reaction (RT-PCR) to observed mRNA expression of GRM2/3/4/6/7/8 subunits by using immunostaining to show the distribution of mGluR2 and mGluR8. The results revealed that the GRM2/3/4/6/7/8 mRNAs were expressed in both rat and human CB. Immunostaining showed that mGluR2 was localized in the type I cells and mGluR8 was localized in type I and type II cells in the rat CB. Moreover, the response of CB to acute hypoxia in rats was recorded by *in vitro* carotid sinus nerve (CSN) discharge. Perfusion of group II mGluRs agonist or group III mGluRs agonist (LY379268 or L-SOP) was applied to examine the effect of group II and III mGluRs on rat CB response to acute hypoxia. We found that LY379268 and L-SOP inhibited hypoxia-induced enhancement of CSN activity. Based on the above findings, group II and III mGluRs appear to play an inhibitory role in the carotid chemoreceptor response to acute hypoxia.

## 1 Introduction

The carotid body (CB), located at the bifurcation of the carotid arteries, serves as a peripheral chemoreceptor for measuring PO_2_, pH, and PCO_2_ (Gonzalez et al., 1994). It consists of glomus or type I cells, which are organized into cell clusters, a rich capillary network, sensory nerve endings, whose somata are in the petrosal ganglion and synaptically connected with type I cell, and sustentacular glial or type II cells distributed around type I cells (Iturriaga et al., 2021). There has been evidence that CB plays an important role in several sympathetic neurodegenerative diseases in humans, including obstructive sleep apnea (OSA), intractable hypertension, congestive heart failure and insulin resistance (Koyama et al., 2000; Prabhakar et al., 2005; Schultz et al., 2007; Iturriaga et al., 2009; Abdala et al., 2012). The mechanisms underlying these diseases that involve CB function have commonly been attributed to changes of cell function trigged by multiple factors such as alterations of the levels of neurotransmitters and neuromodulators in the CB.

Studies have shown that type I glomus cells establish synaptic connections with afferent sensory fibers of the carotid sinus nerve (CSN) as presynaptic neuroendocrine cells (Gao et al., 2019). It is generally accepted that type I cells are the oxygen-transducing cells in the CB, and the mechanism(s) of oxygen transduction likely involve membrane bound O_2_-sensitive potassium channels, which are closed in type I cells under hypoxia, thus leading to membrane depolarization of type I cells (Iturriaga and Alcayaga, 2004). During this process, type I cells release neurotransmitters, and these neurotransmitters act on specific postsynaptic receptors, leading to the excitation of CSN afferent fibers. CB contains multiple neurotransmitters including adenosine triphosphate (ATP), acetylcholine (ACh), norepinephrine (NE), dopamine (DA), etc., and a number of neuromodulators such as angiotensin, endothelin, and nitric oxide (Milsom and Sadig, 1983; Monteiro et al., 2011; Kåhlin et al., 2014). It is reported that hypoxia not only induces CB releasing excitatory transmitters such as ATP, which evokes excitation of the carotid sinus nerve afferents by acting on P2X receptors (Rong et al., 2003), but also triggers CB releasing inhibitory transmitters such as γ -aminobutyric acid (GABA), which binds to presynaptic GABAB receptor to inhibit sensory nerve discharge (Fearon et al., 2003). Although the CB has been shown to contain numerous neurotransmitters and neuromodulators as well as their receptors (Nurse, 2014), there are few reports about glutamatergic signals in the CB.

In the central nervous system (CNS), glutamate is a major neurotransmitter (Zhou and Danbolt, 2014) and is concentrated in presynaptic transmitter vesicles by vesicular glutamate transporters (vGluTs). Glutamate is released into the synaptic space through exocytosis during the depolarization of the presynaptic membrane (Liguz-Lecznar and Skangiel-Kramska, 2007); on the other hand, unbound glutamate in the synaptic space is taken up into astrocytes by excitatory amino acid transporters (EAATs) for further metabolism in order to avoid the excitatory toxicity of glutamate. A remarkable variety of receptors mediate the actions of glutamate in the CNS (Stone, 2021). The fast action of glutamate is mediated by ionotropic glutamate receptors (iGluRs): AMPA, NMDA, and KA-type receptors. These receptors are ligand-gated ion channels that allow fast excitatory synaptic transmission. Metabotropic glutamate receptors (mGluRs) are generally responsible for the slower and long-lasting effects of glutamate and belong to a family of G-protein coupled receptors (GPCRs) that adjust various aspects of neuronal physiology (Remelli et al., 2008; Reiner and Levitz, 2018). mGluRs are classified into three groups: group I mGluRs (mGluR1 and 5), group II mGluRs (mGluR2 and 3), and group III mGluRs (mGluR4, 6, 7, and 8) (Niswender and Conn, 2010). Studies have shown that most group I mGluRs adjusts excitability and plasticity at postsynaptic sites, while group II and III mGluRs mainly act as autoreceptors at presynaptic sites by modulating release properties based on activity. Collectively, presynaptic and postsynaptic mechanisms of transmission and plasticity are functionally coupled by synaptic mGluRs.

Previously, we found that rat CB type I cells expressed vGluT3 and EAAT2/3 (Li et al., 2020), indicating that glutamate may play a role as a neurotransmitter in the CB. Moreover, we found that the functional iGluR subunits NMDAR1 and AMPAR1 exist in rat CB (Liu et al., 2009). Our recent data demonstrated that both the rat CB and human CB express mRNAs of group I mGluR subunits, and that mGluR1 is involved the rat carotid chemoreflex to acute hypoxia via the presynaptic mechanism (Li et al., 2021). In this study, we further explored the mRNA expression of group II and III mGluR subunits in rat and human CB as well as its distribution in rat CB; we mainly focused on mGluR2 and mGluR8, and their role in rat carotid chemoreflex to acute hypoxia response.

## 2 Experimental procedures

### 2.1 Reagents and antibodies

TRIzol reagent was purchased from Thermo Fisher Scientific (Waltham, MA, United States). The HotStarTaq^®^ Master Mix Kit and QuantiTect Reverse Transcription Kit from Qiagen (Valencia, CA, United States). Mouse anti-TH from Sigma (St. Louis, MO, United States). Rabbit anti-mGluR2 was purchased from NOVUS (Littleton, CA, United States). Rabbit anti-mGluR8 was purchased from GeneTex (San Antonio, Texas, United States). Anti-rabbit IgG horseradish peroxidase (HRP)- linked antibodies was purchased from Dingguo Changsheng Biotechnology (Beijing, China). Alexa Fluor 555 goat anti-mouse IgG and mouse anti-GFAP were purchased from Cell Signaling Technology (Danvers, MA, United States). The GTVisionTM III Detection System/Mo & Rb Kit and the 3,3′-diaminobenzidine tetrahydrochloride (DAB) Detection Kit were purchased from Gene Tech (Shanghai, China). O-Phospho-L-serine (L-SOP) was purchased from Selleck (Houston, Texas, United States). (1R,4R,5S,6R)-4-amino-2-Oxabic-yclo [3.1.0] hexane-4,6-dicarboxylic acid (LY379268) was purchased from GLPBIO (Montclair, CA, United States). All primers used in the experiment were synthesized from Beijing Genomics Institute (Shenzhen, China).

### 2.2 Human carotid body harvest

Surgical specimens were approved with the consent of the patient and extracted from The First Affiliated Hospital of Xinxiang Medical University. Human cerebral-cortex tissue was extracted from a patient with craniocerebral trauma and human CB specimen was extracted from a patient with CB paraganglioma. All experimental protocols and surgical procedures were approved by Human Ethics Committee of The First Affiliated Hospital of Xinxiang Medical University.

### 2.3 Animals

Male Sprague-Dawley rats (240–260 g) were used in all animal experiments and ordered from Beijing Vital River Laboratory Animal Technology Co., Ltd. Rats were housed in normal rat cages with light-dark cycle of 12:12-h and were given food and water *ad libitum*. All the procedures in this study were in accordance with the national animal research regulations, and all animal experimental protocols were approved by the Institutional Animal Ethics Committee at The First Affiliated Hospital of Xinxiang Medical University.

### 2.4 Rat carotid body harvest

Rats were anesthetized by inhalation of 2% isoflurane (RWD, Shenzhen, China), and then were decapitated after coma was induced. The carotid bifurcations on two sides were quickly separated and put into phosphate buffered saline (PBS) that was oxygenated (95% O_2_-5% CO_2_) 10 minutes in advance. The CBs were dissected under a stereomicroscope (Nikon SMZ1270, Japan), and were harvested into RNA-later. Sixteen CBs from eight rats were pooled together and stored in -80°C for later use.

### 2.5 RNA extraction and RT-PCR

Total RNA was extracted using the TRIzol reagent, according to the manufacturer’s instructions. cDNA was synthesized through reverse transcription (RT) by QuantiTect Reverse Transcription Kit with 500 ng of total RNA as template in accordance to the manufacturer’s protocol. mRNA expression level was detected by semi-quantitative PCR in a Veriti^®^ 96-well Thermal cycler (Applied Biosystems, Foster City, CA, United States), using HotStarTaq^®^ Master MixKit. To amplify the target gene, 2 μL of cDNA samples was mixed with 10 μL of HotStarTaq Master Mix and 1 μL of gene-specific primer pairs in a final reaction volume of 20 μL. The PCR reactive conditions included an initial step at 95°C for 10 min, followed by 40 cycles at 94°C for 50 s, proper annealing temperature for 50 s, extension at 72°C for 1 min, and then ending at 72°C for 10 min. The PCR products were detected by electrophoresis on a 1.2% agarose gel with tris acetate-EDTA (TAE) and visualized by the gel imaging system UVIpro with the software UVIband (UVItec, Cambridge, United Kingdom). The housekeeping gene β-actin was used as a loading control. Rat or human prefrontal cortex cDNA was used as positive controls. All primers were designed using Primer-BLAST. The sequences of the primers with their transcription product serial number, PCR cycles, and lengths of PCR products are summarized in Tables 1, 2.

**TABLE 1:** Sequence of human primers used in RT-PCR experiment.

Gene	Accession No.	Primer sequence	PCR cycle	Annealing Tm (°C)
*GRM2*	NM_000839.5	F: 5′-CTA​TGG​CGA​GAC​AGG​CAT​TGA-3′	42	55
		R: 5′-CAT​CCT​CAG​AAC​GGG​TGA​ACA-3′		
*GRM3*	**NM_000840.3**	F: 5′-GCA​CCT​CAA​CAG​GTT​CAG​TGT-3′	42	55
		R: 5′-TGG​TGG​AGT​CGA​GGA​CTT​CC-3′		
*GRM4*	**NM_000841.4**	F: 5′-AAT​AAC​CAG​CTG​CGC​AAC​GAT-3′	42	55
		R: 5′-CTG​TCT​TCT​TCC​GCT​CAC​CC-3′		
*GRM6*	**NM_000843.4**	F: 5′-TCC​AGA​CAA​CCA​CGC​TTA​ACC-3′	42	56
		R: 5′-GGG​AGG​AAA​TCT​CCC​GCA​AA-3′		
*GRM7*	**NM_000844.4**	F: 5′-TCC​TTG​CTG​TTG​GAC​CTG​TGA​G-3′	40	57
		R: 5′-CAT​TGT​AGC​GGA​TGA​AAG​TGG​C-3′		
*GRM8*	**NM_001371083.1**	F: 5′-CCA​GAG​CTA​AGT​GAT​AAC​ACC​AG-3′	40	57
		R: 5′-TCT​GTG​ACT​GAG​CAA​TGC​AAA-3′		
*β-actin*	**NM_000005.12**	F: 5′-GCA​GGG​GGG​AGC​CAA​AAG​GGT-3′	30	55
		R: 5′-TGG​TTG​GCA​GTG​ATG​GCA​TGG-3′		

Tm, temperature; F, forward primer; R, reverse primer; NM, reference sequence of mRNA.

**TABLE 2:** Sequence of rat primers used in RT-PCR experiment.

Gene	Accession No.	Primer sequence	PCR cycle	Annealing Tm (°C)
*GRM2*	NM_001105711.1	F: 5′-CTC​CTC​ACC​AAG​ACC​AAT​CG-3′	42	52
		R: 5′-GTG​GTT​ACA​GCG​CAA​TGT​CA-3′		
*GRM3*	**NM_001105712.1**	F: 5′-TAT​TCT​CAG​TCC​TCT​GCA​AG-3′	42	50
		R: 5′-TTG​TAG​CAC​ATC​ACT​ACA​TAC​C-3′		
*GRM4*	**NM_022666.1**	F: 5′-ACA​GTC​AGC​CGA​CAA​GCT​GTA​CAT-3′	42	57
		R: 5′-ATG​GTT​GGT​GTA​GGT​GAC​GTA​GGT-3′		
*GRM6*	**NM_022920.1**	F: 5′-CTG​TTC​CGC​TCT​TCC​TCA​CTT​G-3′	42	55
		R: 5′-ATT​CAG​ACC​TTG​GCT​CAC​CGA​C-3′		
*GRM7*	**NM_031040.1**	F: 5′-ACA​ATT​GGC​GAT​CAC​TTC​CA-3′	42	50
		R: 5′-GTT​CAT​GGT​CTT​ATG​CTC​ATC-3′		
*GRM8*	**NM_022202.1**	F: 5′-AAA​CAA​ACC​GTA​TCC​ACC​G-3′	42	53
		R: 5′-ATC​CCA​GGG​AAC​AAA​TGA​GT-3′		
*β-actin*	**NM_031144.3**	F: 5′-GGG​AAA​TCG​TGC​GTG​ACA​TT-3′	35	53
		R: 5′-CGG​ATG​TCA​ACG​TCA​CAC​TT-3′		

Tm, temperature; F, forward primer; R, reverse primer; NM, reference sequence of mRNA.

### 2.6 Immunohistochemistry staining

The rats were anesthetized with inhalation of 2% isoflurane and fixed via transcardiac perfusion with 4% neutral formalin (Shuangshuang Chemical Co., LTD., Yantai, China). Afterwards, the bifurcation of the carotid artery was separated for paraffin embedding. The paraffin-embedded carotid bifurcation containing the carotid body was sectioned at 3 μm thickness using Shandon™ Finesse™ 325 Microtomes (Thermo Fisher Scientific, Waltham, United States). The prepared sections of the rat carotid body were placed at 65°C and roasted for 1 h, followed by xylene I and II dewaxing for 10 min each. Then, the sections were washed and rehydrated with gradient alcohol (100%, 95%, 80%, and 60%) for 3 min each. After washing with PBS for 3 times, 5 min each time, the tissue sections were immersed in 0.01 M sodium citrate buffer solution (pH 6.0) for antigen retrieval at 100°C for 15 min. The slices were then cooled down and washed 3 times with PBS. To quench the activity of tissue endogenous peroxidase, the slices were immersed in 3% H_2_O_2_ at room temperature for 10 min, and then were permeabilized with 0.2% Triton X-100 solution for 15 min. After being blocked with 10% goat serum at 37°C for 1 h, sections were incubated overnight at 4°C with the following primary antibodies: rabbit anti-mGluR2 (1:50, NOVUS, Cat No. NBP2-67900) and rabbit anti-mGluR8 (1:300, GeneTex, Cat No.108159). The next day, sections were cleaned three times in PBS and incubated with GTVisionTM III Detection System Mouse & Rabbit Kit, according to the manufacturer’s instruction. The sections were treated with an anti-rabbit/mouse HRP-labeled secondary antibody for 30 min at 37°C, then reacted with DAB, and then stained for further microscopic analysis. Paraffin-sectioned the sections of brain hippocampus area were used as positive control, while the negative control was primary antibody replaced with PBS. The nucleus was stained using hematoxylin. Finally, the sections were dried naturally and the cover slips were applied onto the slides, which were sealed with neutral adhesive. The images were observed with the Nikon H600L microscope and photographed with Nikon digital camera DS-Fi1c (Nikon, Tokyo, Japan).

### 2.7 Double immunofluorescence staining

Double immunofluorescence staining was performed to examine the localization of mGluR2 and mGluR8 in rat CB. The protocols prior to incubation of primary antibody were the same as immunohistochemistry without 3% H_2_O_2_ incubation. 5% bovine serum albumin was used to dilute the primary antibodies as listed: monoclonal rabbit anti-mGluR2 (1:50) and mGluR8 antibodies (1:300), monoclonal mouse anti-TH (1:1000, Sigma, Cat No. T2928), and anti-GFAP antibody (1:200, CST, Cat No. 3670). Each anti-mGluR2/8 antibody plus anti-TH or GFAP was individually applied to the sections overnight at 4°C. The following secondary antibodies were used: Alexa Fluro 488 goat anti-rabbit IgG (1:300, CST, Cat No. 4412S) and Alexa Fluro 555 goat anti-mouse IgG (1:300, CST, Cat No. 4409S). The staining was examined and photographed with the Nikon C2 confocal microscope (Nikon, Tokyo, Japan).

### 2.8 *Ex vivo* electrophysiological recording of the carotid sinus nerve (CSN) activity.

Rats were deeply anesthetized with 2% isoflurane and carotid bifurcations were removed *en bloc*. The carotid bifurcations were placed into a Petri dish containing ice-cold Krebs’ solution (in mM: NaCl 113, KCl 5.9, NaH_2_PO_4_ 1.2, MgSO_4_ 1.2, NaHCO_3_ 25, Glucose 11.5, CaCl_2_ 2, and pH 7.4) bubbled with carbogen (95% O_2_-5% CO_2_). The CSN was dissected carefully and cleaned from surrounding connective tissue. Then, the CB-CSN preparation was placed into the recording chamber (volume = 6.5 ml) and a catheter was placed at each end of the chamber to bring the infusion in and out. The recording chamber was maintained at 36°C and perfused at a flow rate of 15 ml/min with Krebs’ solution bubbled with 5% CO_2_ balance in O_2,_ for a pH of 7.4. The CSN is sucked into the tip of the glass electrode for activity recording. The electrode tip was fully inhaled (double check correct verb) and sealed to prevent connective tissue from surrounding the junction of the CB and the CSN. Meanwhile, a grounding electrode was placed into the recording chamber. The neural signal was fed into a head-stage filtered (100–1,000 Hz) differential input, and amplified (1,000 ×) by DP-311 differential amplifier (Warner Instruments, Hamden, CT, United States). The signal was then processed by PowerLab 8/35 (AD Instruments, Bella Vista, New South Wales, Australia) and analyzed and integrated by LabChart 8 software (AD Instruments, Bella Vista, New South Wales, Australia).

### 2.9 Experimental protocol and statistical analysis

CB-CSN preparation was perfused with Krebs’ solution bubbled with 95% O_2_-5% CO_2_, referred to as “baseline solution,” and CSN discharge was also recorded to establish a baseline. Once getting stably responsive discharge of CSN to acute hypoxia, the following 3 steps were performed. First, the CB-CSN preparation was perfused with Krebs’ solution equilibrated with a hypoxic gas mixture (5% O_2_-5% CO_2_-90% N_2_), named as “hypoxia solution,” for 90 s to evoke the CSN firing; then, the perfusate was switched to baseline solution for 10 min wash out. Second, the preparation was perfused with baseline solution containing group II mGluRs agonist (1R,4R,5S,6R)-4-amino-2-oxabicyclo [3.1.0] hexane-4,6-dicarboxylic acid (LY379268, 100 nM), or group III mGluRs agonist O-Phospho-L-serine (L-SOP, 200 µM) for 5 min, followed by superfusion with hypoxia solution containing the agonist mentioned about for 90 s; then, the washing out was carried out by superfusion with baseline solution for 15 min. Finally, the third hypoxic infusion was performed for 90 s before perfusate was replaced with baseline solution for 15 min. All solutions were delivered at a rate of 15 ml/min, and CSN activity was assessed by measuring the number of impulses above threshold per second on a second-by-second basis. All statistical analyses were performed using SPSS 23.0. All data were normally distributed by Levene’s test, and the data were presented as mean ± SEM. Statistical evaluation was conducted by One-Way ANOVA with repeated measures. *p* < 0.05 was considered statistically significant.

## 3 Results

### 3.1 mRNA expression of group II and III mGluRs in human and rat CB

**FIGURE 1:**
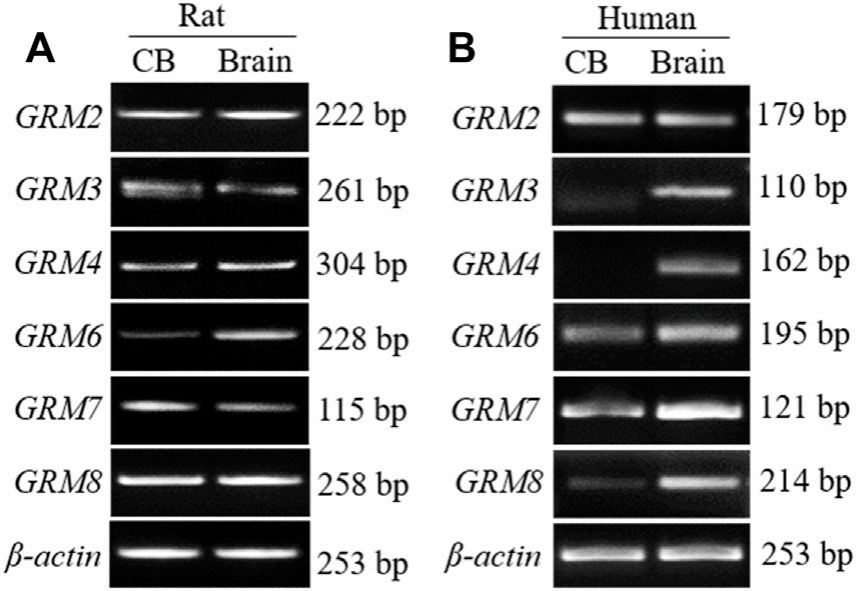
mRNA expression of group II and III mGluRs in rat and human CB. **(A,B)** Representative agarose gel images show the RT-PCR products of group II and III mGluR subunit in rat and human CB (CB, left lanes in **(A,B)**. RNA extracted from rat or human prefrontal cortex was used as positive control (Brain, right lanes in **(A,B)**. CB, carotid body.

We have reported that mRNAs of group I mGluR (*GRM1/5*) are expressed in human CB as well as rat CB (Li et al., 2021). To further determine whether group II (*GRM2/3*) and group III (*GRM/4/6/7/8*) subunits are expressed in the CB, we examined the mRNA expression of those mGluR subunits by RT-PCR, using specific primers that recognize unique sequences of each mGluR subtype. As shown in Figure 1, PCR-amplified transcripts corresponding to the predicted sizes of group II and III mGluRs were shown in rat (Figure 1A) and human CB (Figure 1B) as well as in the rat cortex and human brain tissue, both of which were used as the positive controls. In rat CB, all PCR products of group II and III mGluR subunits were detected, the intensity of *GRM2* transcript was stronger than that of *GRM3* in group II mGluRs, and the intensity of *GRM8* was clearly stronger than that of other subunits in group III mGluRs. Based on the results, the expression trend of some genes, were different between human CB tested in this study and the rat CB, i.e. the intensity of GRM8 transcript band was much weaker in this human CB than that in rat CB, and GRM4 PCR product was undetectable in human CB but clearly visible in rat CB. These data give us heuristic that the mRNAs of group II and III mGluR subunits maybe widely expressed in the CB with some different expression trends among these mRNAs between rat and human CB.

### 3.2 Distribution of mGluR2 and mGluR8 in the rat CB

**FIGURE 2:**
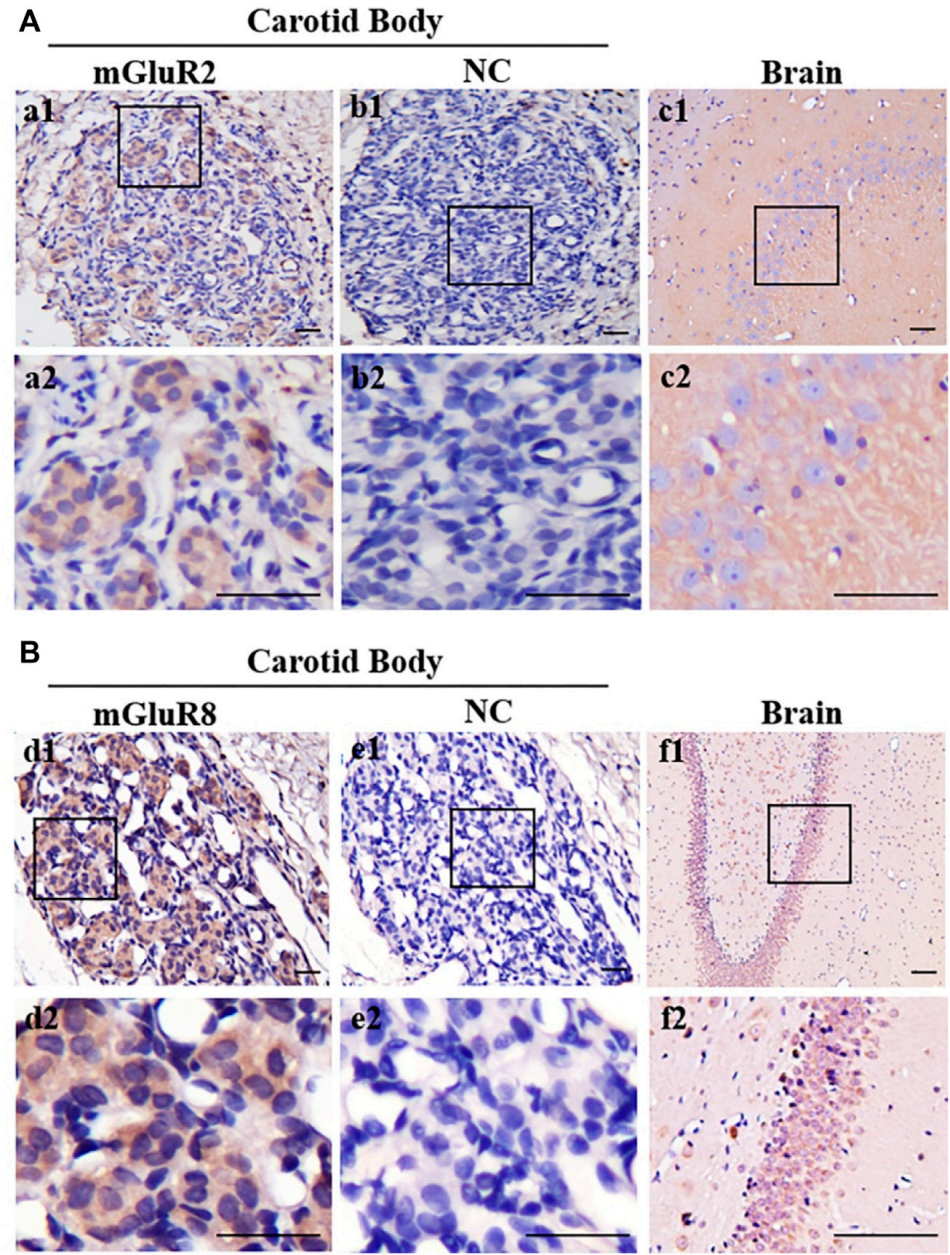
Immunohistochemical staining of mGluR2 and mGluR8 in rat CB. The representative images of rat CB and brain sections were immunostained with mGluR2 **(A)** and mGluR8 **(B)**. The brown staining represents the mGluR2 (Figure a1) or mGluR8 (Figure d1), while the blue staining represents hematoxylin-stained cell nuclei. Figure b1 and e1 are negative staining controls obtained by omitting the primary antibody. Figure c1-2 and f1-2 of rat brain hippocampus area are individually positive controls for mGluR2 and mGluR8. Figure a2-f2 are higher magnification images of framed areas in a1-f1, respectively. mGluR: glutamate metabotropic receptor; NC: negative control. Scale bar = 50 μm for all images.

In terms of the RT-PCR results, mGluR2 and mGluR8 were further characterized in the distribution of rat CB *via* immunohistochemistry. As shown in Figures 2A,B, positive immunoreaction towards both mGluR2 (Figure a1) and mGluR8 (Figure d1) was observed in rat CB, and the negative control (Figure b1 and e1) obtained by omitting the primary antibody did not yield specific staining in rat CB. Rat brain sections with intense immunoreactivity were used as positive controls (Figure c1 and f1). Immunostaining for mGluR2 and mGluR8 were diffusely distributed in the CB, but mainly in organized cell clusters. These data further support the presence of mGluR2 and mGluR8 in rat CB.

### 3.3 Cellular localization of mGluR2 and mGluR8 in the rat CB

**FIGURE 3:**
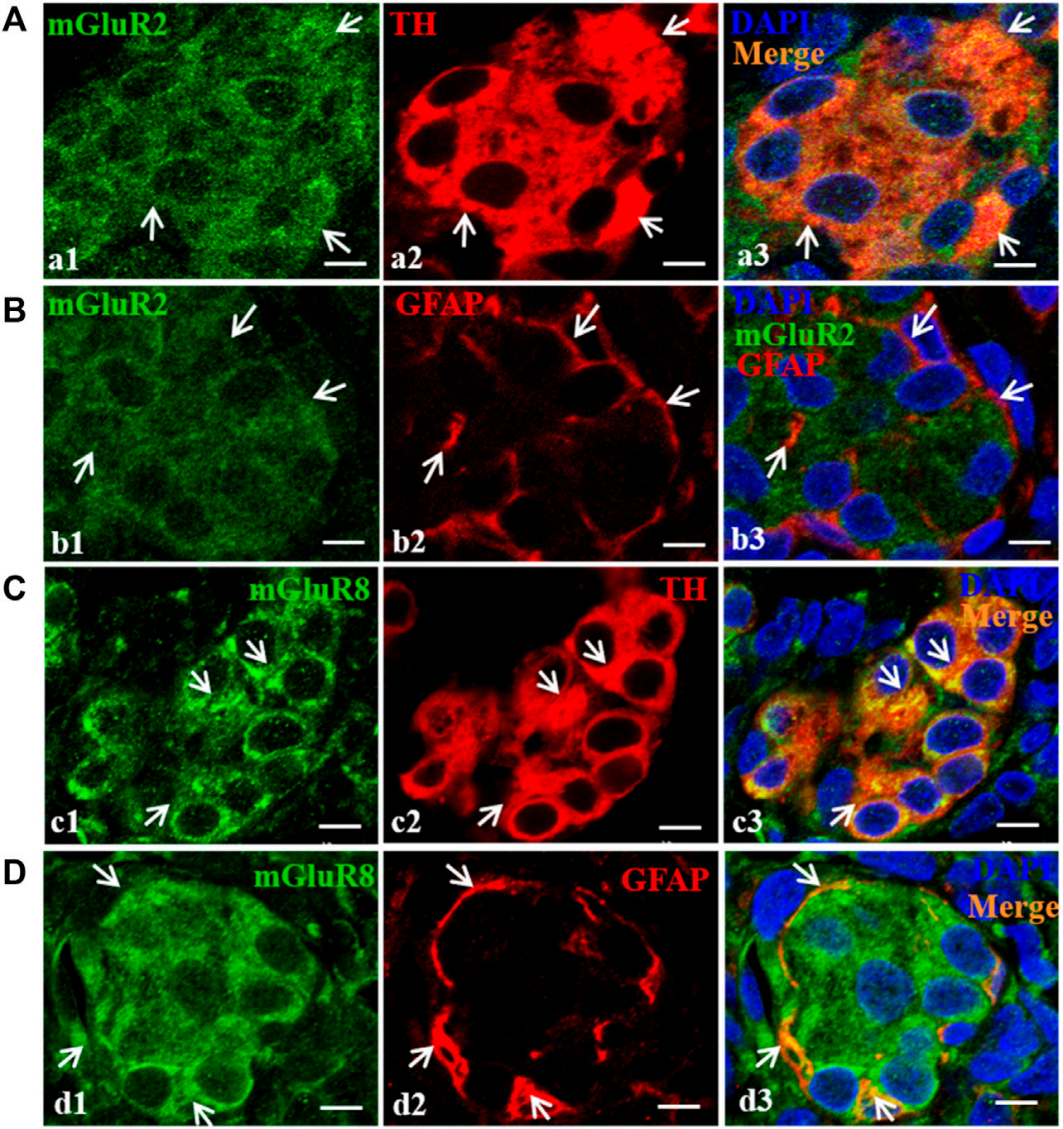
Distribution of mGluR2 and mGluR8 in rat CB. Figure **(A)** and **(C)** are double immunofluorescence staining of mGluR2 (Figure a1) or mGluR8 (Figure c1, green) with TH (Figure a2 or c2, red) in rat CB. Figure **(B)** and **(D)** are double immunofluorescence staining of mGluR2 (Figure b1) or mGluR8 (Figure d1, green) and GFAP (Figure b2 or d2, red) in rat CB. Some TH-immunoreactive type I cells are immunoreactive for mGluR2 (Figure a3, arrowheads), whereas the GFAP-immunoreactive type II cell are not immunoreactive for mGluR2 (Figure b3). Some TH-immunoreactive type I cells and the GFAP-immunoreactive type II cells were immunoreactive for mGluR8 (Figure c3 and d3, arrowheads). TH, tyrosine hydroxylase, the CB type I cell marker; GFAP, glial fibrillary acidic protein, the CB type II cell marker; mGluR, glutamate metabotropic receptor. Scale bar = 5 μm for all images.

To further ascertain the cellular localization of mGluR2 and mGluR8 in rat CB, double immunofluorescence staining was performed. In the CB, type I glomus cells are usually recognized by labeling tyrosine hydroxylase (TH), and type II sustentacular cells are identified by glial fibrillary acidic protein (GFAP). Double immunofluorescence of mGluR2 with TH or GFAP were performed in consecutive sections of the rat CB. As shown in Figures 3A,B, mGluR2 immunoreactive signals were clearly observed in TH-positive glomus type I cells (Figure a3), but were not merged with GFAP-labeled type II cells (Figure b3), indicating that mGluR2 is distributed in type I cells rather than in type II cells in rat CB. Double immunofluorescence of mGluR8 with TH or GFAP in consecutive sections, as shown in Figures 3C,D, revealed that mGluR8 immunoreactive signals were clearly observed in both TH-positive glomus type I cells (Figure c3) and GFAP-positive type II cells (Figure d3), indicating that mGluR8 is expressed in type I and II cell in rat CB.

### 3.4 Activation of group II or III mGluRs attenuates CB response to hypoxia

**FIGURE 4:**
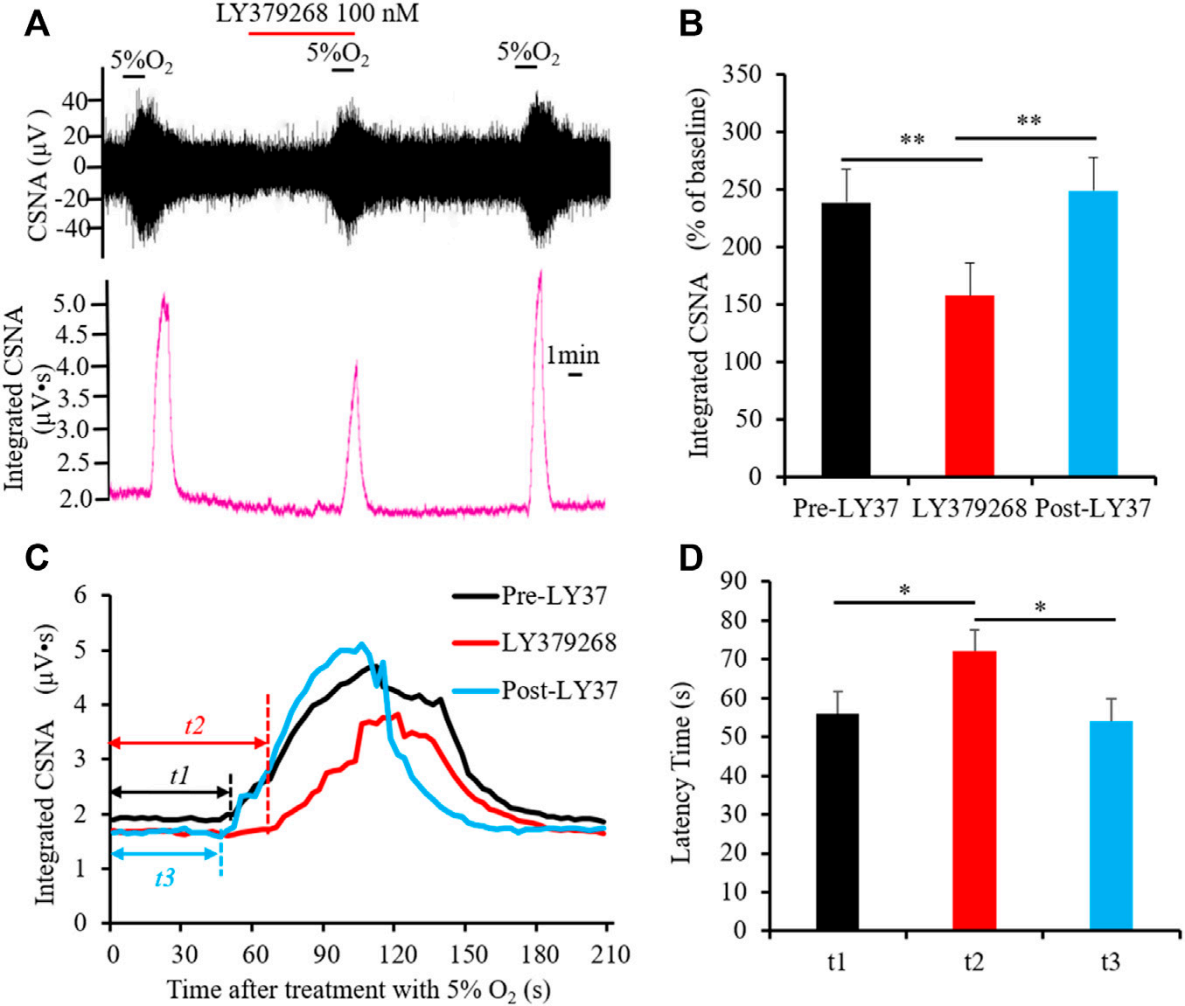
LY379268 attenuates CB response to hypoxia. **(A,B)** LY379268 (100 nM) inhibited hypoxia-evoked CSN discharge. **(A)** Shows the representative recordings of CSN discharge and integrated CNSA before, after, and post treatment of LY379268 (Pre-LY37, LY37 and Post-LY37); **(B)** reveals the data analysis of hypoxia-evoked integrated CSNA, represented as the percentage of baseline. **(C,D)** LY379268 extended the duration of hypoxia-evoked CSN firing. *t* represents the latency time that is referred to as the time from superfusion hypoxia solution to the onset of CSN sharply firing. *t1*: Latency time of CB response to hypoxia before treatment with LY379268 (Pre-LY37); *t2*: Latency time of CB response to hypoxia while treated with 100 nM LY379268 (LY37); *t3*: Latency time of CB response to hypoxia after wash out LY379268 (Post-LY37). **(C)** Shows the representative recordings of integrated CNSA in the condition of Pre-LY37, LY37, and Post-LY37; **(D)** reveals the data analysis of the latency times of CB response to hypoxia in the condition of Pre-LY37, LY37, and Post-LY37. LY37: represents LY379268 and group II mGluRs agonist; CSNA: carotid sinus nerve activity. The data were presented as mean ± SEM. *n* = 4, **p < 0.01 and *p < 0.05 indicate significant difference (One-Way ANOVA with repeated measures).

**FIGURE 5:**
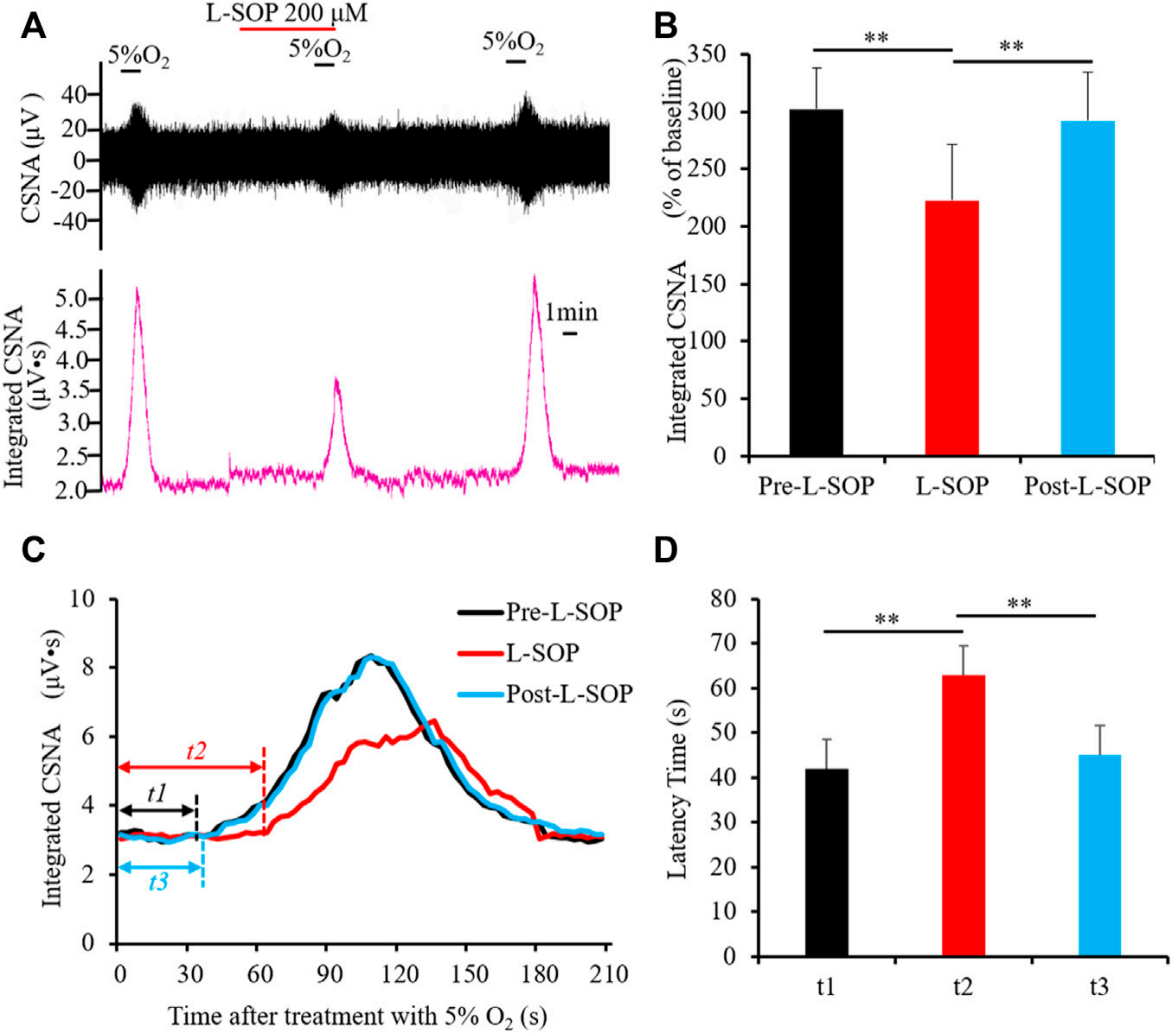
L-SOP attenuates CB response to hypoxia. **(A,B)** L-SOP (200 µM) inhibited hypoxia-evoked CSN discharge. **(A)** Shows the representative recordings of CSN discharge and integrated CNSA before, after, and post treatment of L-SOP (Pre- L-SOP, L-SOP and Post- L-SOP); **(B)** reveals the data analysis of hypoxia-evoked integrated CSNA, represented as the percentage of baseline. **(C,D)** L-SOP extended the duration of hypoxia-evoked CSN firing. *t* represents the latency time that is referred to as the time from superfusion hypoxia solution to the onset of CSN sharply firing. *t1*: Latency time of CB response to hypoxia before treatment with L-SOP (Pre- L-SOP); *t2*: Latency time of CB response to hypoxia while treatment with 200 μM L-SOP (L-SOP); *t3*: Latency time of CB response to hypoxia after wash out with L-SOP (Post- L-SOP). **(C)** Shows the representative recordings of integrated CNSA in the condition of Pre- L-SOP, L-SOP, and Post- L-SOP; **(D)** reveals the data analysis of the latency times of CB response to hypoxia in the condition of Pre- L-SOP, L-SOP, and Post- L-SOP. L-SOP: Group III mGluRs agonist; CSNA: carotid sinus nerve activity. The data were presented as mean ± SEM. *n* = 6, **p < 0.01 indicate significant difference (One-Way ANOVA with repeated measures).

As a peripheral chemoreceptor, the CB senses the reduction of arterial PO_2_, leading to the afferent activation of CSN, which is also known as the CB response to hypoxia. To investigate the effect of group II or III mGluRs on the CB response to hypoxia, CSN discharge was recorded by *in vivo* electrophysiology. A representative trace of carotid sinus nerve activity (CSNA) was shown in Figures 4A, 5A. Similar to a previous study (Rong et al., 2003), the CSN discharge increased sharply after superfusion with the hypoxia-saturated buffer. Of note, application of either group II mGluR agonist LY379268 (100 nM) or group III mGluR agonist L-SOP (200 µM) into basal perfusate (95% O_2_-5% CO_2_ saturated Kreb’s solution) significantly lowered the CSN discharge evoked by hypoxia (normalized integrated CSNA before vs. after treatment with LY379268 238.78 ± 19.13 vs. 165.27 ± 13.44%, *n* = 4, *p* < 0.01, Figures 4A,B, before vs. after treatment with L-SOP 302.92 ± 10.51 vs. 219.18 ± 14.85%, *n* = 6, *p* < 0.01, Figures 5A,B). The time from exposure to hypoxia to the onset of CSNA increase is referred to as latency time (t, Figures 4C, 5C) of CB response to acute hypoxia. As shown in Figure 4, the latency times of CB response to hypoxia before, after, and post treatment of group II mGluR agonist LY379268 were 56.28 ± 3.72 s, 72.39 ± 4.34 s, and 54.36 ± 4.35 s (Figures 4C,D, *n* = 4, *p* < 0.05). As shown in Figure 5, latency time of CB response to hypoxia before, after, and post treatment of group III mGluR agonist L-SOP were 42.35 ± 2.28 s, 63.21 ± 2.32 s, and 45.12 ± 3.24 s (Figures 5C,D, *n* = 6, *p* < 0.01). Both LY379268 and L-SOP extended the duration of hypoxia-evoked CSN firing. These results demonstrate that activation of group II or III mGluRs attenuates the CB response to hypoxia.

## 4 Discussion

The current data demonstrate that mRNAs of group II mGluRs (GRM2/3) and group III mGluRs (GRM/4/6/7/8) subunits are expressed in the rat and human CB. The data on mGluR2 and mGluR8 reveal that immunoactivity of mGluR2 and mGluR8 is present in rat CB. mGluR2 localizes to type I cell and mGluR8 is present in both type I and type II cells in the CB. Activation of group II or III mGluR inhibits CB response to acute hypoxia. According to our review of published literature, this is the first study demonstrating functional group II and III mGluRs existing in rat CB.

Although the CB has been demonstrated to contain a number of neurotransmitters and neuromodulators, as well as its receptors (Nurse, 2014), there are few reports on glutamatergic signals in the CB. Previously, glutamate in the CB was considered a metabolite rather than a neurotransmitter since perfusion of the CB with high concentration of potassium solution did not cause the release of glutamate from the cat CB (Torrealba et al., 1996). However, our group found that vGluTs, which are considered to be specific biomarkers that identify glutamate as a neurotransmitter, exist in both human CB and rat CB, and that vGluT3, a major type of vGluTs in rat CB, is mainly distributed in type I cells. These findings indicate that glutamate might function as a neurotransmitter in the CB. Our previous studies also found multiple iGluR subunits existing in rat CB. Recently, RNAseq results from Pauza and colleagues (Pauza et al., 2022) show that multiple iGluRs are expressed in rat CB and that iGluR levels are upregulated in spontaneously hypertensive rat CB compared with wild type rat CB, providing further evidence for glutamatergic signaling in the CB.

MGluRs are divided into group I mGluR (mGluR1 and 5), group II mGluR (mGluR2 and 3), and group III mGluR (mGluR4, 6, 7, and 8), and play a critical role in the synaptic transmission in and plasticity of the CNS. We have recently reported that mRNAs of group I mGluR subunits are expressed in human and rat CB, and that mGluR1 is distributed in rat type I cells and attenuates rat CB response to acute hypoxia through potential presynaptic mechanisms (Li et al., 2021). In this current study, we further demonstrated that mRNAs of group II and III mGluR subtypes are diversely expressed in rat and human CB (Figure 1). Human CB specimen from a single patient with CB paraganglioma was used in this study. Considering its limitation on the source and sample size, the expression pattern of mGluRs in human CB is needed to further validate by means of more human CB samples.

Type I cells function as sensory elements in the CB to establish chemosensory synapses with afferent CSN fibers and to work as presynaptic neurosecreting elements that transduce chemical signals during afferent nerve discharge in the solitary tract nucleus. These functions ultimately cause sympathetic nerve excitation (Porzionato et al., 2019). There are synaptic structures between adjacent type I cells as oxygen sensing cells or interneurons are interconnected by synapses and an afferent nerve ending of the IXth cranial nerve (IXth) forms reciprocal synapses with a type I A cell (Morgan et al., 1975; Krammer, 1978), indicating the presence of synaptic transmission and a complex modulating loop between type I cells. Type II cells that are GFAP-expressing glia-like cells with interleaved processes envelope the type I cell clusters, and not only play a supporting role for type I cells, but also affect synaptic neurotransmission from type I cells to the CSN terminal via paracrine mechanisms (Tse et al., 2012; Zhang et al., 2012). In this study, based on the strength of the transcript products, the distribution of mGluR2 in group II mGluR and mGluR8 in group III mGluR was further explored (Figures 2A,B). mGluR2 and mGluR8 are diffusely present in rat CB; mGluR2 localizes in type I cells rather than type II cells (Figures 3A,B), whereas mGluR8 is not only distributed in type I cells, but also in type II cells (Figures 3C,D). Therefore, both mGluR2 and mGluR8 might affect carotid chemoreflex response via presynaptic mechanisms. mGluR8, due to its distribution feature in rat CB, might also be responsive to a more subtle mechanism to modulate rat carotid chemoreflex response.

It has been demonstrated that group II mGluRs are located predominantly on presynaptic terminals and serve as inhibitory receptors to regulate synaptic transmission (Pin and Duvoisin, 1995; Conn and Pin, 1997; Scanziani et al., 1997; Cartmell and Shoepp, 2000). For instance, Shigemoto et al. (1997) reported that mGluR2 immunoreactivity was located primarily in the presynaptic terminal of the hippocampus. Several physiological and biochemical studies in rodent prefrontal cortex (PFC) have found that activation of group II mGluRs localized in the presynaptic membrane reduces glutamate release and decreases excitation of the postsynaptic neuron (Kamiya et al., 1996; Moghaddam and Adams, 1998; Benneyworth et al., 2007; Wang et al., 2012). The studies also show that blocking group II mGluRs in the PFC increases the extracellular level of glutamate (Jin et al., 2017), implying that group II mGluRs negatively modulate neuron synaptic transmission *via* presynaptic prevention of glutamate release. Group II mGluRs also influence synaptic transmission by regulating other neurotransmitters. In cultured rat cortical primary neurons, application of LY354740, a group II agonist, inhibited KCl-induced GABA release (Schaffhauser et al., 1998). Electrophysiological recording showed that activation of group II mGluRs receptors reduced the amplitude of GABA-mediated inhibitory postsynaptic currents in the CA1 region of the hippocampus (Liu et al., 1993). By using *in vivo* microdialysis, Miyamoto et al. found that overexpression of Shati/Nat8l, a N-acetyltransferase, in mouse nucleus accumbens (NAc) activated group II mGluRs, thereby attenuating methamphetamine-induced elevation of extracellular dopamine (Miyamoto et al., 2014). This finding implies a restrictive role of group II mGluRs on DA release in the NAc. Jensen et al. (2006) determined that, in the presence of ambenonium (an acetylcholinesterase inhibitor), group II mGluRs inhibited the release of the neurotransmitter Ach from starburst amacrine cells. On the basis of previous reports, group II mGluRs mainly play an inhibitory effect on neurotransmission by modulating multiple neurotransmitters. In this study, we explored the function of group II mGluRs in the rat carotid chemoreflex response to acute hypoxia, and demonstrated that activation of group II mGluRs attenuated the acute hypoxia-induced discharge of CSN, in altitude (Figures 4A,B) and in latency time (Figures 4C,D). Type I cells contain a large number of neurotransmitters and neuromodulators, such as ATP, DA, Ach, etc. Moreover, we found vGluT3 in type I cells, implying that glutamate functions as a neurotransmitter in type I cells. Based on the presence of mGluR2 distribution in type I cells in rat CB and the characteristics of group II mGluRs, we speculate that, by modulating the presynaptic release of glutamate and/or other neurotransmitters, activation of group II mGluRs in rat CB may blunt hypoxia signal transmission from type I cells to CSN discharge.

Group III mGluRs are extensively expressed in the CNS and their localization have been found in both presynaptic axons and postsynaptic membranes of different CNS regions (Bradley et al., 1996). Activation of group III mGluRs modulates neurotransmission by affecting multiple types of neurons. Awad-Granko H et al. reported that activation of group III mGluRs by application of L-AP4 in rat subthalamic nucleus (STN) presynaptically suppressed GABAergic synaptic transmission by reducing GABA release. In comparison, L-AP4 in rat STN-substantia nigra pars reticulate (SNr) synapse presynaptically reduced glutamatergic synaptic transmission by blocking the glutamate release from glutamatergic terminals (Bonci et al., 1997). Activation of group III mGluRs may also have different effects on glutamate release in different regions. For instance, Evans et al. found that activation of group III mGluRs in layer II of the entorhinal cortex (EC) resulted in depressed glutamate release; however, activation of group III mGluRs in layer V facilitated the spontaneous excitatory postsynaptic current (sEPSC) by presynaptically prompting glutamate release (Evans et al., 2000). Based on these aforementioned findings, the role of activation of group III mGluRs on neurotransmission might be dependent on its location and the effect of which neurotransmitter is released, i.e. glutamate or GABA. In this study, we found that application of L-SOP, an agonist of group III mGluRs, attenuated the firing activity of hypoxia-induced CSN discharge (Figures 5A,B), and also prolonged the latency time of CB response to hypoxia (Figures 5C,D). Based on the characteristics of group III mGluRs and our data, we hypothesized that activation of group III mGluRs presynaptically attenuates hypoxia-evoked carotid chemoreflex by inhibiting glutamate release and/or facilitating GABA release from type I cells. Additionally, type II cells might be involved in this attenuation process.

One of the limitations in this study is that the agonists used were not group II or III mGluR subunit-specific. In the following study, we will further verify the function of mGluRs, especially mGluR2 and mGluR8, in the CB by using specific group II or III mGluR subunit agonists. In addition, the combination of the agonist with the antagonist of groups II or III mGluRs is needed to further elucidate the functions of these receptors in the process of the CB response to hypoxia. The mechanisms by which group II and III mGluRs attenuate the CB chemoreflex to hypoxia also need clarification.

In summary, our new finding reveals that functional groups of II and III mGluRs are present in the CB. mGluR2, a subunit of group II mGluRs, is distributed in type I cells of rat CB, and mGluR8, a subunit of group III mGluRs, is distributed in both type I and type II cells of rat CB. Activation of group II or III mGluRs attenuates rat CB response to hypoxia.

## Data Availability

The datasets presented in this study can be found in online repositories. The names of the repository/repositories and accession number(s) can be found in the article/Supplementary Material.

## Abbreviations

ATP, adenosine triphosphate; AMPARs, alpha-amino-3-hydroxy-5-methyl-4-isoxazolepropionic acid receptors; Ach, acetylcholine; CSN, carotid sinus nerve; CB, carotid body; DA, dopamine; EAATs, excitatory amino acid transporters; GABA, γ -aminobutyric acid; GFAP, glial fibrillary acidic protein; GPCRs, G-protein coupled receptors; iGluRs, inotropic glutamate receptors; KA, Kainate; L-SOP, O-Phospho-L-serine; LY379268, (1R,4R,5S,6R)-4-amino-2-Oxabic-yclo [3.1.0] hexane-4,6-dicarboxylic acid; mGluRs, metabotropic glutamate receptors; NMDARs, N-methyl-D-aspartate receptors; NE, norepinephrine; TH, tyrosine hydroxylase; vGluTs, vesicular glutamate transporters.
